# Drug interaction between tacrolimus and nilotinib in a patient with chronic myeloid leukemia after renal transplant

**DOI:** 10.1002/ccr3.900

**Published:** 2017-03-17

**Authors:** Takashi Onaka, Naoto Takahashi, Masatomo Miura, Akihito Yonezawa

**Affiliations:** ^1^ Department of HematologyKokura Memorial HospitalKitakyushuJapan; ^2^ Department of Hematology, Nephrology, and RheumatologyAkita University Graduate School of MedicineAkitaJapan; ^3^ Department of PharmacyAkita University HospitalAkitaJapan

**Keywords:** Chronic myeloid leukemia, nilotinib, pharmacokinetics, renal transplant, tacrolimus

## Abstract

Nilotinib, a BCR‐ABL tyrosine kinase inhibitor, is a known inhibitor of CYP3A4 and could increase the concentration of drugs metabolized by CYP3A4. An immunosuppressive drug for nilotinib‐treated patients following transplant should be administered with careful pharmacokinetic monitoring because of its interaction with nilotinib.

## Case Examination

**Figure 1:**
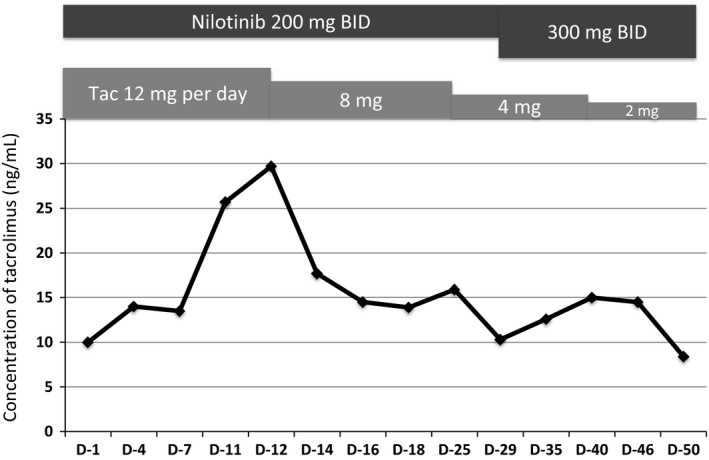
Concentration of tacrolimus increased transiently because of its interaction with nilotinib in a patient with chronic myeloid leukemia after renal transplantation.

A 49‐year‐old woman on maintenance hemodialysis (HD) for polycystic kidney disease was diagnosed as having chronic phase Philadelphia‐positive chronic myeloid leukemia (CML). Initially, she was administered 300 mg imatinib once per day; however, a major molecular response (MMR, BCR‐ABL1 transcript level ≤0.1%) was not achieved even after 2 years of imatinib treatment. Although the standard dose of nilotinib in imatinib‐resistant disease is 400 mg twice a day (BID), she was administered 200 mg nilotinib BID because she had CKD, which is a risk factor for cardiovascular adverse events. However, the only adverse event related to 200 mg nilotinib BID was muscle pain (grade 2). An MMR was achieved 6 months after switching to nilotinib. Four years later, she underwent cadaveric renal transplantation and continued 200 mg nilotinib BID. Maintenance HD was not necessary on day 9 after renal transplantation. We had to decrease tacrolimus dose by 34% (from 12 to 8 mg/day) due to a transient increase in its blood concentration (Fig. 1). After adjusting the dosage of tacrolimus, no major side effects were observed, including renal dysfunction. One month after renal transplantation, plasma nilotinib concentration was measured by high‐performance liquid chromatography 1, which revealed that the nilotinib trough concentration was lower than the mean trough concentration in nilotinib phase 1/2 trial (348 ng/mL vs. 615 ng/mL) 2. There was no adverse event associated with nilotinib, and the concentration of tacrolimus was also within the therapeutic range (8–12 ng/mL). She was therefore administered 300 mg nilotinib BID. Although we had to decrease tacrolimus dose again (Fig. 1), the plasma nilotinib concentration increased to the optimal range (948 ng/mL) without any adverse events (Table 1), and a deep molecular response (BCR‐ABL1 transcript level ≤0.0032%) was achieved 34 months after cadaveric renal transplantation.

**Table 1:** Pharmacokinetics of nilotinib after renal transplant

Dose of nilotinib	200 mg BID	300 mg BID
Time after renal transplant	1 month	2 months
Serum creatinine level	1.28 mg/dL	1.44 mg/dL
Plasma trough concentration of nilotinib	348 ng/mL	948 ng/mL

BID, twice a day.

## Discussion

Nilotinib is a second‐generation BCR‐ABL inhibitor used to treat imatinib‐resistant/intolerant CML. The safety and efficacy of nilotinib have been reported in previous clinical trials 3, 4, 5. An optimum plasma trough concentration of nilotinib is important for maximum efficacy in patients with imatinib‐resistant or intolerant CML 2. However, some patients with renal dysfunction or at risk for drug interactions may not tolerate standard nilotinib doses. Previously, we reported the nilotinib plasma concentrations during maintenance HD and suggested that neither is plasma nilotinib removed by HD nor is its pharmacokinetics influenced by HD 6. However, the appropriate nilotinib dosage for renal transplant recipients has not been reported. As expected, our study suggests that nilotinib pharmacokinetics is not altered in renal transplant recipients, as compared to that in patients without renal dysfunction, as neither metabolism nor excretion of nilotinib occurs via the two kidneys. However, the expected interaction between nilotinib and tacrolimus leads to an increase in the blood concentration of tacrolimus due to the inhibitory action of nilotinib against CYP3A4 and P‐glycoprotein 7, 8, similar to the results reported for the patient in this study. Our results indicate that the standard dose of nilotinib can be safely administered but tacrolimus administration needs careful pharmacokinetic monitoring in transplant patients because of the drug interaction with nilotinib.

## Authorship

TO: managed the patient and wrote the manuscript. NT: analyzed data and wrote the manuscript. MM: analyzed data, and AY: reviewed the manuscript.

## Conflict of Interest

NT received research funding and a speaker's bureau from Pfizer, Novartis and Otsuka. The other authors declare no competing interests.
